# Supplementary material for: Conditional chemoconnectomics (cCCTomics) as a strategy for efficient and conditional targeting of chemical transmission
Source: eLife. 2024 Apr 30;12:RP91927. doi: 10.7554/eLife.91927 (PMC11060718; doi:10.7554/eLife.91927)
Supplement: Figure 3—source data 3. [file elife-91927-fig3-data3.zip › Figure 3 source data3 part 1.zip!/Figure 3-Source data 3-part1/1082-9-CRISPResso_Report_zod2ewbs.zip!/CRISPResso_on_1082_10/9.Alleles_frequency_table_around_sgRNA_catccaggcagatccaggtg.pdf]

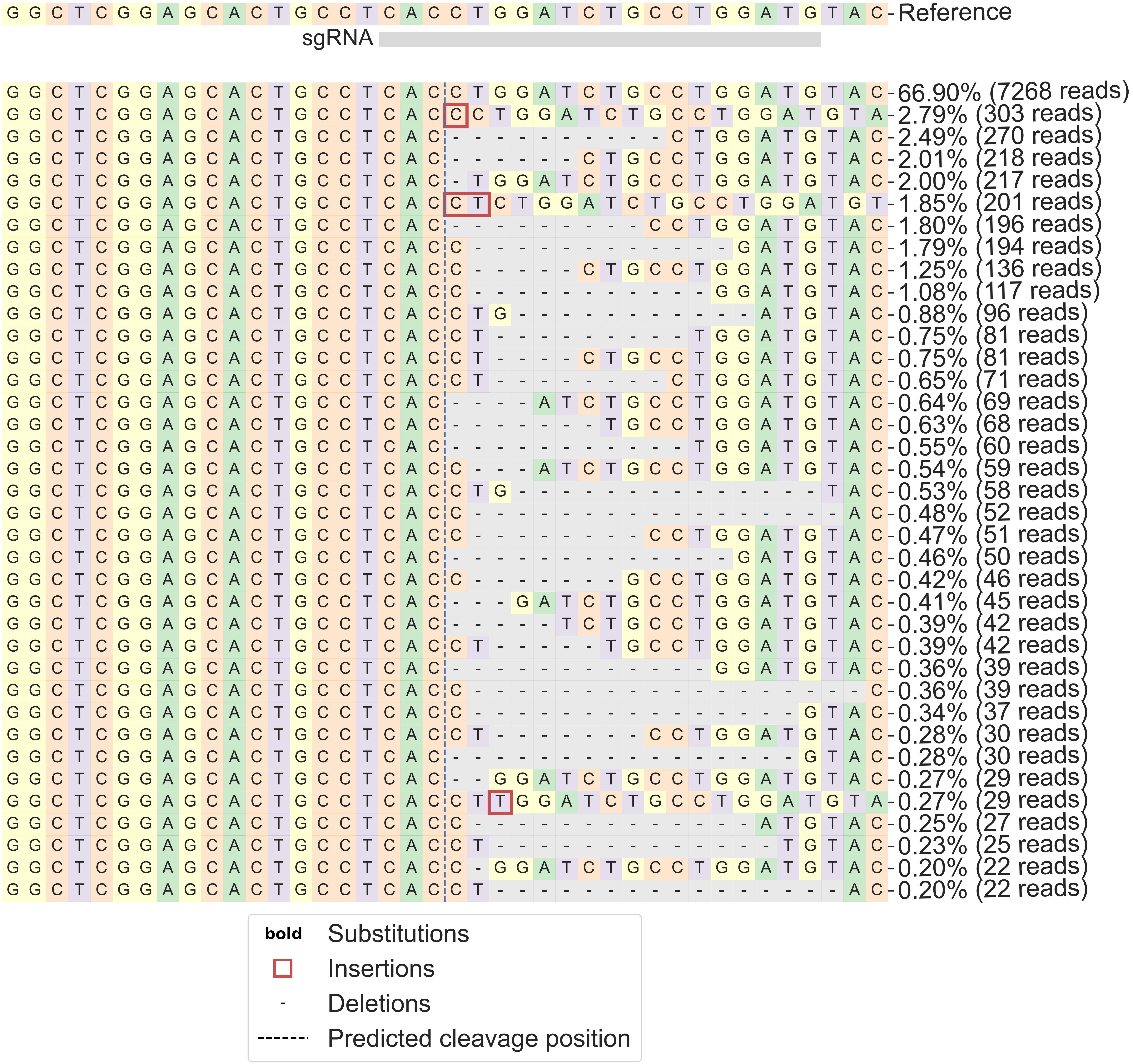

GGCTCGGAGCACTGCCTCACCTGGATCTGCCTGGATGTAC Reference
sgRNA
GGCTCGGAGCACTGCCTCACCTGGATCTGCCTGGATGTAC 66.90% (7268 reads)
GGCTCGGAGCACTGCCTCACCCTGGATCTGCCTGGATGTA 2.79% (303 reads)
GGCTCGGAGCACTGCCTCAC----------CTGGATGTAC 2.49% (270 reads)
GGCTCGGAGCACTGCCTCAC------CTGCCTGGATGTAC 2.01% (218 reads)
GGCTCGGAGCACTGCCTCAC-TGGATCTGCCTGGATGTAC 2.00% (217 reads)
GGCTCGGAGCACTGCCTCACCTCTGGATCTGCCTGGATGT 1.85% (201 reads)
GGCTCGGAGCACTGCCTCAC---------CCTGGATGTAC 1.80% (196 reads)
GGCTCGGAGCACTGCCTCACC------------GATGTAC 1.79% (194 reads)
GGCTCGGAGCACTGCCTCACC-----CTGCCTGGATGTAC 1.25% (136 reads)
GGCTCGGAGCACTGCCTCACC-----------GGATGTAC 1.08% (117 reads)
GGCTCGGAGCACTGCCTCACCTG-----------ATGTAC 0.88% (96 reads)
GGCTCGGAGCACTGCCTCACCT---------TGGATGTAC 0.75% (81 reads)
GGCTCGGAGCACTGCCTCACCT----CTGCCTGGATGTAC 0.75% (81 reads)
GGCTCGGAGCACTGCCTCACCT--------CTGGATGTAC 0.65% (71 reads)
GGCTCGGAGCACTGCCTCAC----ATCTGCCTGGATGTAC 0.64% (69 reads)
GGCTCGGAGCACTGCCTCAC-------TGCCTGGATGTAC 0.63% (68 reads)
GGCTCGGAGCACTGCCTCAC-----------TGGATGTAC 0.55% (60 reads)
GGCTCGGAGCACTGCCTCACC---ATCTGCCTGGATGTAC 0.54% (59 reads)
GGCTCGGAGCACTGCCTCACCTG--------------TAC 0.53% (58 reads)
GGCTCGGAGCACTGCCTCACC-----------------AC 0.48% (52 reads)
GGCTCGGAGCACTGCCTCACC--------CCTGGATGTAC 0.47% (51 reads)
GGCTCGGAGCACTGCCTCAC-------------GATGTAC 0.46% (50 reads)
GGCTCGGAGCACTGCCTCACC-------GCCTGGATGTAC 0.42% (46 reads)
GGCTCGGAGCACTGCCTCAC---GATCTGCCTGGATGTAC 0.41% (45 reads)
GGCTCGGAGCACTGCCTCAC-----TCTGCCTGGATGTAC 0.39% (42 reads)
GGCTCGGAGCACTGCCTCACCT-----TGCCTGGATGTAC 0.39% (42 reads)
GGCTCGGAGCACTGCCTCAC------------GGATGTAC 0.36% (39 reads)
GGCTCGGAGCACTGCCTCACC------------------C 0.36% (39 reads)
GGCTCGGAGCACTGCCTCACC---------------GTAC 0.34% (37 reads)
GGCTCGGAGCACTGCCTCACCT-------CCTGGATGTAC 0.28% (30 reads)
GGCTCGGAGCACTGCCTCAC----------------GTAC 0.28% (30 reads)
GGCTCGGAGCACTGCCTCAC--GGATCTGCCTGGATGTAC 0.27% (29 reads)
GGCTCGGAGCACTGCCTCACCTTGGATCTGCCTGGATGTA 0.27% (29 reads)
GGCTCGGAGCACTGCCTCACC-------------ATGTAC 0.25% (27 reads)
GGCTCGGAGCACTGCCTCACCT-------------TGTAC 0.23% (25 reads)
GGCTCGGAGCACTGCCTCACC-GGATCTGCCTGGATGTAC 0.20% (22 reads)
GGCTCGGAGCACTGCCTCACCT----------------AC 0.20% (22 reads)
bold Substitutions
Insertions
Deletions
Predicted cleavage position
